# Calendar

**DOI:** 10.1080/15604280214935

**Published:** 2002


*Int. Jnl. Experimental Diab. Res.*, 3:265, 2002
Copyright © 2002 Taylor & Francis
1560-4284/02 $12.00 + .00
DOI: 10.1080/15604280290014017

Taylor & Francis healthsciences

### CALENDAR

**June 13–17, 2003**
**63rd Scientific Sessions**
**New Orleans, LA**
Location: New Orleans Convention Center
Sponsorship: American Diabetes Association

Contact information for ADA events: American Diabetes Association, 1701 N. Beauregard St., Alexandria, VA 22311.
Phone: 800-232-3472, select option 5.
Meeting information fax-on-demand:
703-299-5513 or 703-683-1351.
e-mail: meetings@diabetes.org

**June 17–21, 2003**
**New Genetic and Metabolic Insights into Animal Models of Diabetes**
**Bar Harbor, Maine**
Abstract Deadline: March 1, 2003

Registration Inquiries should be directed to:
Judi Alexander
Lead Coordinator,
Courses and Conferences
The Jackson Laboratory
600 Main Street
Bar Harbor, ME 04609
Phone: 207-288-6326
Fax: 207-288-6080
e-mail: judih@jax.org

Please check out our web site
http://www.jax.org/courses/
for the current listing of courses and conferences at The Jackson Laboratory.

**30 August–September 2, 2003**
**6th Diabetic Neuropathy Satellite Symposium to the 18th International Diabetes Federation Congress in conjunction with NEURODIAB XIII - 13th Annual Meeting of the Diabetic Neuropathy Study Group of the EASD**
**Saint Malo, France**
This symposium follows the tradition of the previous five triennial Diabetic Neuropathy IDF Satellite meetings that have been held in San Jose (Costa Rica) in 2000, Noordwijkerhout (The Netherlands) in 1997, Hakone (Japan) in 1994, New York (USA) in 1991, and Singapore in 1988.

The aim is to give physicians and researchers from all over the world the opportunity to exchange their views and ideas on the putative mechanisms underlying the various, potentially devastating neuropathic complications and to discuss how they could be detected at earliest stages and most effectively treated or prevented.

Deadline for submission of abstracts: 1st March 2003

For registration please contact:
Agence EVIC
58, rue Trousseau
75011 Paris, France
Tel: +33-1-4923 0117
Fax: +33-1-4923 8019
e-mail: camillevoisin@evicevents.com

**May 26–29, 2004**
**13th European Congress on Obesity (ECO-Prague)**
**Prague Congress Centre, Prague, Czech Republic**
Main topics include:
- Epidemiology
- Cellular Mechanisms and Pathophysiology
- Genetics of Obesity
- Health and Social Consequences
- Management
- Prevention

For further information please contact the Congress Organizer:
Guarant Ltd., Opletalova 22
CZ-11000 Prague 1
Czech Republic
Tel: +42-2-84001444
Fax: +42-2-84001428
e-mail: eco@guarant.cz
http://www.eco2004.cz

